# Tanshinone I alleviates motor and cognitive impairments via suppressing oxidative stress in the neonatal rats after hypoxic-ischemic brain damage

**DOI:** 10.1186/s13041-017-0332-9

**Published:** 2017-11-14

**Authors:** Chunfang Dai, Yannan Liu, Zhifang Dong

**Affiliations:** 1 Ministry of Education Key Laboratory of Child Development and Disorders, Children’s Hospital of Chongqing Medical University, 136 Zhongshan Er Road, Yuzhong District, Chongqing, 400014 People’s Republic of China; 2 Chongqing Key Laboratory of Translational Medical Research in Cognitive Development and Learning and Memory Disorders, Children’s Hospital of Chongqing Medical University, 136 Zhongshan Er Road, Yuzhong District, Chongqing, 400014 People’s Republic of China; 3 China International Science and Technology Cooperation base of Child Development and Critical Disorders, Children’s Hospital of Chongqing Medical University, 136 Zhongshan Er Road, Yuzhong District, Chongqing, 400014 People’s Republic of China

**Keywords:** Hypoxic-ischemic brain damage, Tanshinone I, Learning and memory, Oxidative stress

## Abstract

Neonatal hypoxia-ischemia is one of the main reasons that cause neuronal damage and neonatal death. Several studies have shown that tanshinone I (TsI), one of the major ingredients of Danshen, exerts potential neuroprotective effect in adult mice exposed to permanent left cerebral ischemia. However, it is unclear whether administration of TsI has neuroprotective effect on neonatal hypoxic-ischemic brain damage (HIBD), and if so, the potential mechanisms also remain unclear. Here, we reported that treatment with TsI (5 mg/kg, i.p.) significantly alleviated the deficits of myodynamia and motor functions as well as the spatial learning and memory in the rat model of HIBD. These behavioral changes were accompanied by a significant decrease in the number of neuronal loss in the CA1 area of hippocampus. Moreover, ELISA assay showed that TsI significantly increased the production of antioxidants including total antioxidant capacity (T-AOC), glutathione (GSH), total superoxide dismutase (T-SOD) and catalase (CAT), and reduced the production of pro-oxidants including hydrogen peroxide (H_2_O_2_), total nitric oxide synthase (T-NOS) and inducible nitric oxide synthase (iNOS). Taken together, these results indicate that TsI presents potential neuroprotection against neuronal damage via exerting significantly antioxidative activity and against pro-oxidant challenge, thereby ameliorating hypoxia-ischemia-induced motor and cognitive impairments in the neonatal rats, suggesting that TsI may be a potential therapeutic agent against HIBD.

## Introduction

Neonatal hypoxic-ischemic encephalopathy (HIE), which is caused by perinatal hypoxia-ischemia, is one of the major reasons that lead to neuronal damage and neonatal death. The incidence of HIE is about 1 to 3 per 1000 term births [1, 2], and up to 40,000 to 50,000 infants are affected each year in China. The survivor exhibits motor disability or a variety of serious neurological sequela, such as cerebral palsy, epilepsy, severe learning impairment or intellectual deficiency [3–5], which diminish the quality of life of HIE children and increase the enormous burden of economic and spirit on their families and society. Therefore, the research for a potential neuroprotective therapy in order to guide the clinical treatment for HIE is particularly important.

Recent studies have proposed that oxidative injury to vital cellular structures contributes to the pathogenesis of HIE [6]. The potential mechanism underlying oxidative injury in HIE is that the neonatal brain is selectively vulnerable to oxidative stress, which results in altered reactive oxygen species metabolism [7] including the increased accumulation of hydrogen peroxide and subsequent neurotoxicity [8]. In addition, the brain of neonatal is immature and the nervous system exerts immature antioxidant defenses, which display less activity in antioxidant enzyme systems including superoxide dismutase (SOD) and glutathione peroxides [9, 10]. There is a growing body of evidence has shown that oxidative stress results in calcium mobilization and cell damage via providing the link between activation of glutamate receptors and the intracellular cascade of events. Those findings explain the appearance of delayed cell death and secondary energy failure, suggesting that the inhibition of oxidative stress may be a potential therapeutic for HIE [11].

Danshen (Radix salvia miltiorrhiza root), an annual sage plant, is among the most popular medicinal herbs used in China, whose extract as an antihypertensive and a sedative is widely used for the treatment of cardiovascular and cerebrovascular diseases in recent years [12–14]. The water-soluble Danshen extracts contain several diterpene quinine analogs including tanshinone I (TsI), tanshinone II (TsII), cryptotanshinone (CTs) and dihydrotanshinone I (DTsI) [15], which are able to penetrate the blood-brain barrier [16]. Given its antioxidantive and anti-inflammatory effects both in vitro [17–19] and in vivo [19–21], tanshinone is of particular therapeutic interest in hypoxic-ischemic brain damage (HIBD). Indeed, both TsI and TsII including TsIIA and TsIIB, have obvious potential for neuroprotection against hypoxia-ischemia injury in adult mice and rat [22–24]. In addition, treatment with TsIIA significantly reduced the severity of brain injury induced by hypoxia-ischemia in the immature rat [25, 26]. However, there is no any report on the influence of TsI on motor and cognitive functions in neonatal rat after HIBD.

As aforementioned, TsI has potential anti-oxidative activity. We therefore hypothesize that TsI may present neuroprotective effects through suppressing oxidative stress, and subsequently improve motor function and learning and memory ability in neonatal rat model of HIBD. In the present study, we investigated this hypothesis using a combination of behavioral test, immunohistochemical and anti-oxidative activity analysis in the neonatal rat model of HIBD.

## Methods

### Experimental animals

Unsexed 7-day-old Sprague-Dawley (SD) rats were used to establish the HIBD model as previously described, with modification [27]. Briefly, the left carotid artery of 7-day postnatal rats was ligated and 2 h later the pups were exposed to hypoxic conditions (8% O_2_ + 92% N_2_) for 2.5 h at 37 °C. The sham animals were only separated out the left carotid artery without ligation and no exposure to hypoxic conditions. Immediately after hypoxic treatment, pups were transferred back to the nest with their dam until weaning on postnatal day 21, and they were housed in plastic cages with unlimited access to food and water and maintained in a temperature-controlled colony room (21 °C) under a cycle of 12-h light/12-h dark (8:00 am - 8:00 pm).

### TsI treatment

**Fig. 1:**
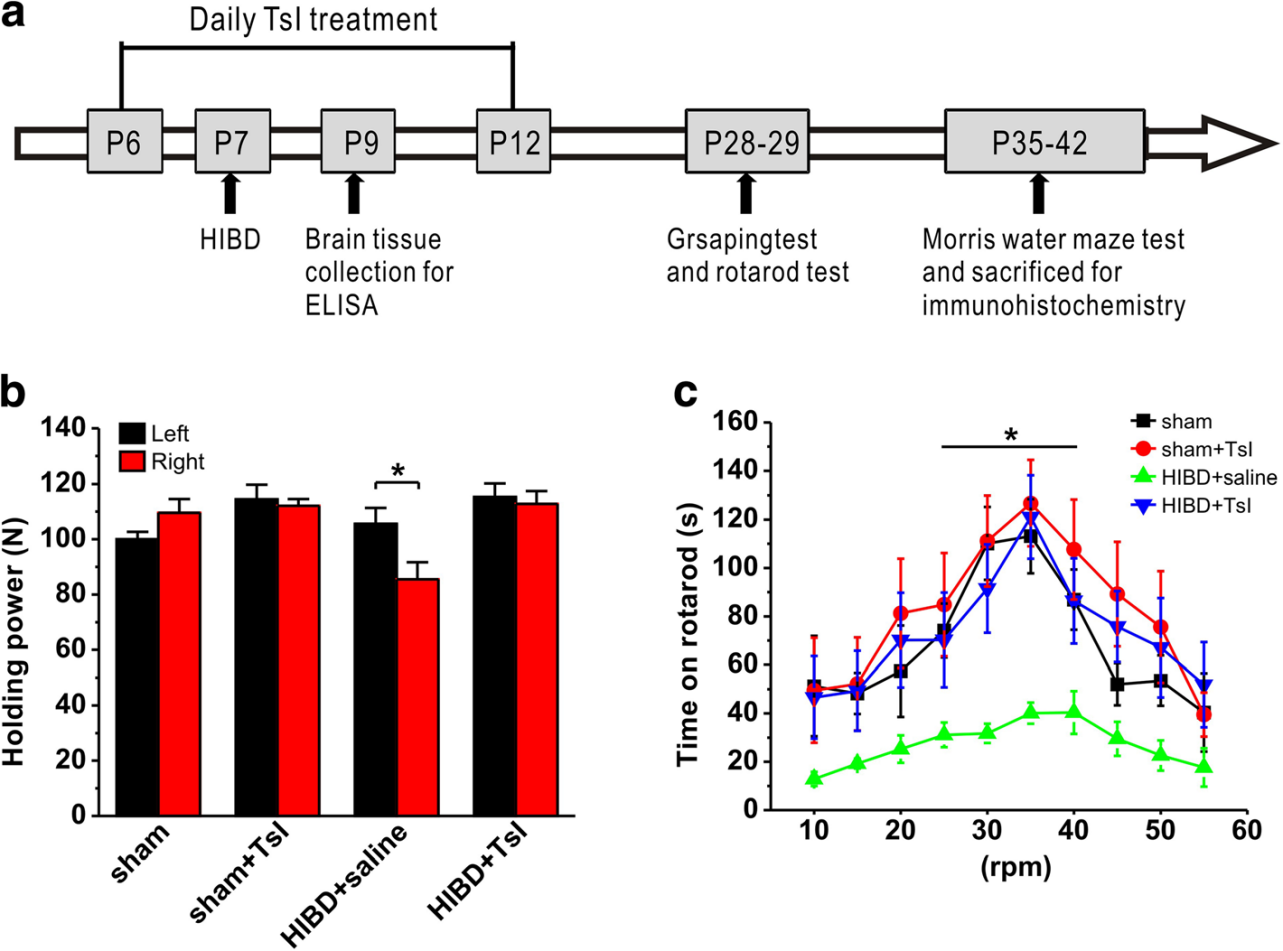
Pretreatment of TsI rescues the deficits of myodynamia and motor functions in neonatal HIBD rats. **a** The flowchart illustrates the experimental protocols of TsI treatments and behavioral tests. Rats received TsI treatment (5 mg/kg per day, i.p.) twice before and five times after HIBD model establish, and the second treatment of TsI was 6 h before the surgery. Two days after HIBD, brain tissue was collected for oxidative stress analysis. Grasping test and rotarod test were performed on postnatal day 28 and 29 (P28–29), and the Morris water maze test was performed from postnatal day 35 to 42 (P35–42). After behavioral tests, brain tissues were collected for immunohistochemistry. **b** The left and right forelimb myodynamia during the grasping test. **c** The latency to fall off the rod during the rotarod test. The data was presented as mean ± SEM. *p < 0.05

To illustrate the neuroprotective effects of TsI against HIBD, all pups were divided into four groups: sham, sham + TsI, HIBD + saline, HIBD + TsI. TsI was purchased from selleck (Shanghai, China) and dissolved in sterile saline containing 0.5% dimethyl sulfoxide (DMSO). The rats in sham + TsI and HIBD + TsI groups received intraperitoneal (i.p.) injection of TsI at a dose of 5 mg/kg/day from 1 day before hypoxic-ischemic surgery (postnatal day 6) for 7 days, and the second injection was conducted 6 h before the surgery (Fig. 1a), as reported previously [23, 26].

To further examine the therapeutic effects of TsI against HIBD, some rats received 7-day treatment of TsI (5 mg/kg/day, i.p.) from postnatal day 7 to 13, and the first injection was conducted immediately after HIBD (Fig. 3a).

### Grasping test

Grasping test was used to evaluate myodynamia according to the instruction (Chatillon, USA). Briefly, 3 weeks after HIBD model established, the left and right forelimbs of each rat were placed on the grasping force sensor, respectively. Gently pulled the trail and recorded the last maximum tensile force until the rat cannot hold on. The myodynamia of left or right forelimb was measured 5 times and mean value was calculated to illustrate the myodynamia of the rats. The Grip Strength Meter was cleaned with 70% ethanol and water between tests.

### Rotarod test

Rotarod test was used to evaluate motor performance as previously described, with modifications [28]. Briefly, after grasping test, each rat received 2 rounds of pre-training trials (one trial at 0 rpm and the other trial at 20 rpm) on the rotarod (Stoelting Co.). Twenty-four hours after the pre-training, rats were received formal rotarod test in ten consecutive trials (20-min intervals) with an initial rotation of 5 rpm to last rotation 50 rpm (increasing by 5 rpm each time). The time that the animals remained on the rotarod during each test was monitored, and maximum test time (cut-off limit) was 180 s. The latency to fall off the rotarod was used to express the motor performance. The rotarod was cleaned with 70% ethanol and water between tests.

### The Morris water maze test

Four weeks after HIBD, the Morris water maze test was performed to measure spatial learning and memory as described previously [29, 30]. In brief, rats were allowed to adapt to the water maze, which consists of a circular pool (180-cm diameter, 60-cm at the side), for 60 s 24 h before the first spatial learning trial. During training period, rats were trained in the pool over 4 trials per day for 5 consecutive days to find a submerged platform (1-cm below the surface of the water). During each trial, the rats failed to reach the hidden platform within 60 s were guided to the platform where they remained for 30 s. A 60-s probe test was performed without the hidden platform to assess the memory retrieval ability, 24 h after the last training trial. All trials were recorded by using Any-maze tracking system (Stoelting, USA).

### Immunohistochemistry

After behavioral tests, the rats were deeply anesthetized with urethane (1.5 g/kg, i.p.) and then transcardially perfused with 0.9% saline followed by 4% paraformaldehyde in 100 mM phosphate-buffered saline (PBS, PH 7.4). Brains were transferred to 30% sucrose in 100 mM PBS for several days before they sunk to the bottom of sucrose solution. Then they were serially sectioned into 30-μm coronal sections using Leica cryostat and every sixth slice with the same reference position was stained. After blocking and permeabilization, the slices were incubated with diluted anti-NeuN (1:100 dilution, Millipore, MAB377) for overnight at 4 °C. Thereafter, the positive neurons were visualized with anti-mouse Ig HRP detection kit according to the manufacturer’s instruction. The number of NeuN-positive cells was quantified in brain sections with typical morphology of the pyramidal neuron in the CA1 area of hippocampus. Briefly, the number of NeuN-immunoreactive neurons was counted manually at five-section intervals throughout the CA1 area of hippocampus by bright-field microscopy using ImageJ software. To quantify changes of the number of NeuN-immunoreactive neurons in the CA1, the number of NeuN-immunoreactive neurons in sham rat was normalized to 100%, and the number of NeuN-immunoreactive neurons in other groups was expressed as a percentage of the sham.

### Oxidative stress analysis

Brain tissues of hippocampus and cortex were homogenized with saline (tissue weight: saline volume = 1: 9) on ice, followed by centrifugation at 2500 g for 10 min at 4 °C. The supernatant was used to test the antioxidant activity including total antioxidant capacity (T-AOC), glutathione (GSH), superoxide dismutase (SOD) and catalase (CAT), and pro-oxidants including hydrogen peroxide (H_2_O_2_), total nitric oxide synthase (TNOS) and inducible nitric oxide synthase (iNOS), by using ELISA kit (Nanjing Jiancheng Bioengineering Institute, Nanjing, China). Briefly, the supernatant of brain tissue homogenate was mixed with Reagent 1 (the volume of supernatant: Reagent 1 = 1: 1) by vortex, and then centrifuged at 3500 g at 4 °C for 10 min. Then, 100 μl mixed tissue sample was pipetted into a well of microplate, and 100 μl Reagent 2 and 25 μl Reagent 3 were added to each well and incubated at room temperature for 5 min. The absorbance was determined at 405 nm by microplate reader (Bio Tek Insruments). Finally, the standard curve was used to determine the concentration of each sample.

### Statistical analysis

All data were expressed as means ± standard error (mean ± SEM). The differences of rotarod test and spatial learning in water maze test among different groups were analyzed by two-way AVOVA with treatment (group) as the between-subjects factor and training trials in rotarod test or learning day in water maze test as the within- subjects factor. The data of all other experiments were analyzed by one-way ANOVA followed by Tukey’s post hoc test. Significance level was set as at *p* < 0.05.

## Results

### TsI ameliorates myodynamia and motor deficit in HIBD rat

To determine whether TsI can rescue HIBD-induced motor deficits, two different behavioral tests were introduced: grasping test and rotarod test. In grasping test, myodynamia of the right forelimb was significantly decreased compared to that of the left in HIBD rats (HIBD + saline: *n* = 10, Fig. 1b). Importantly, treatment with TsI (5 mg/kg, i.p.) fully rescued the HIBD-induced myodynamia deficit, as reflected by similar myodynamia in both left and right forelimbs (HIBD + TsI: *n* = 11, Fig. 1b). Notably, myodynamia was not affected with or without TsI treatment in sham groups (sham: n = 10; sham + TsI: n = 10, Fig. 1b).

In rotarod test, the rats in HIBD group spent much less time on the rod compared with those treated with sham surgery (sham: *n* = 8; HIBD + saline: n = 8, *p* < 0.05 vs. sham; Fig. 1c), indicating a significant impairment of motor balance and coordination. Treatment with TsI fully rescued the HIBD-induced motor deficit, as reflected by a dramatic increase in the time spent on the rod (HIBD + TsI: *n* = 9, *p* > 0.05 vs. sham, p < 0.05 vs. HIBD + saline; Fig. 1c), whereas TsI treatment have no effect on motor function in the sham group (sham + TsI: n = 8, p > 0.05 vs. sham; Fig. 1c). Taken together, these results indicate that TsI treatment alleviates the deficits of myodynamia and motor function in the neonatal rat after HIBD.

### TsI ameliorates spatial learning and memory in HIBD rats

**Fig. 2:**
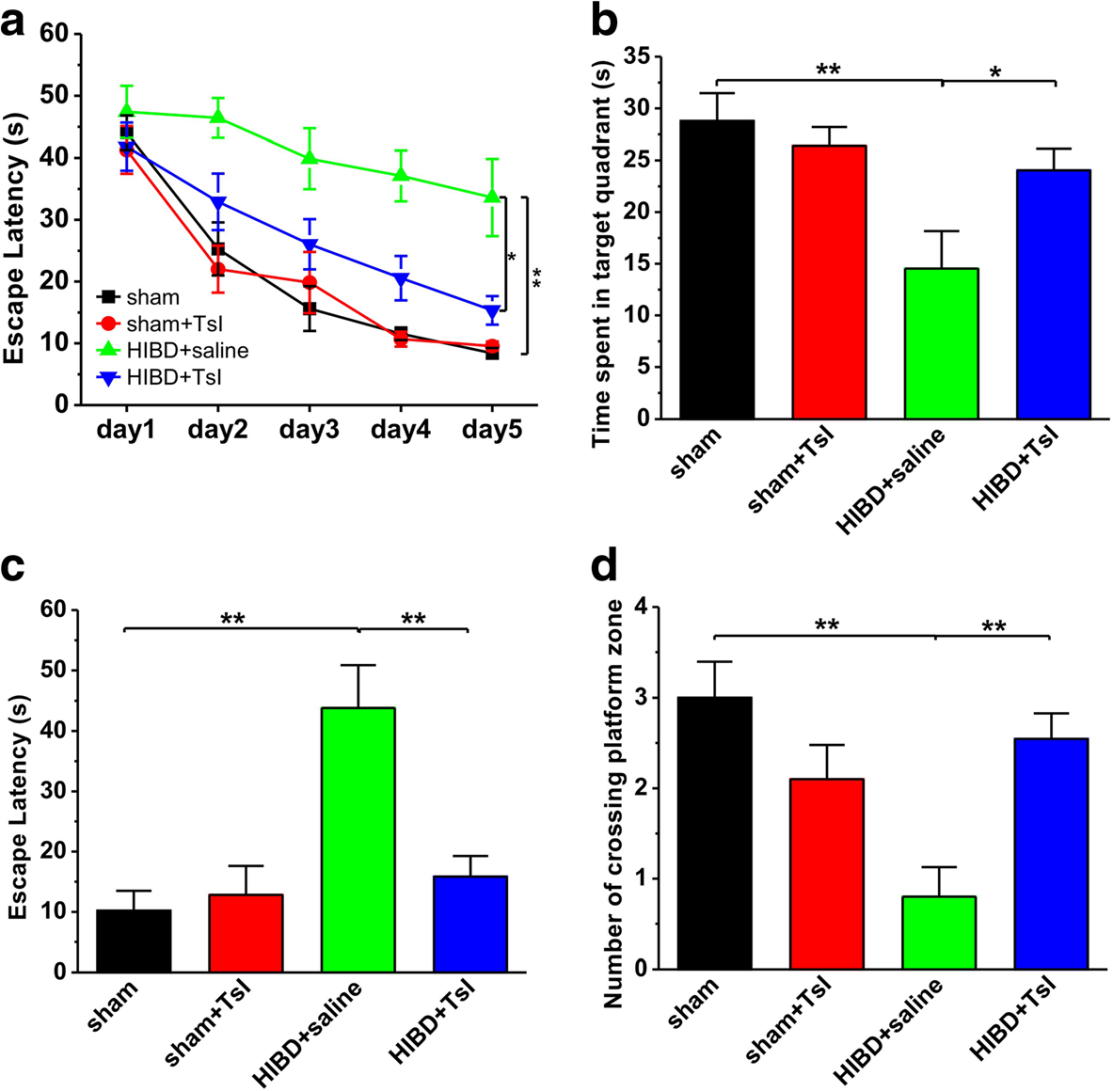
Pretreatment of TsI alleviates spatial learning and memory deficits in neonatal HIBD rats. **a** The average escape latency to the hidden platform location is plotted for each spatial learning day in the Morris water maze task. **b**-**d** Bar graph showed the time spent in the hidden platform-located quadrant **b**, the latency of first time to the hidden platform location (**c**) and the number of entries into the platform zone (**d**) during the probe test with absence of the hidden platform, which is conducted 24 h after the last learning trial. Data was expressed as mean ± SEM. *p < 0.05, **p < 0.01

It has been well documented that the impairment of learning and memory is the major sequelae of HIBD in human and a variety of animal models [31, 32]. To identify the effects of TsI treatment on spatial learning and memory deficits induced by hypoxia-ischemia in neonatal rats, the Morris water maze test, a hippocampus-dependent task, was conducted. As shown in Fig. 2, although the escape latency decreased progressively in all groups, the latency in the HIBD group was much longer than that in the sham groups during spatial training period (sham: *n* = 10; sham + TsI: *n* = 10, *p* > 0.05 vs. sham; HIBD + saline: n = 10, *p* < 0.01 vs. sham; Fig. 2a), indicating an impairment of learning after HIBD. Importantly, daily TsI treatment (5 mg/kg, i.p.) significantly ameliorated the impairment, as reflected by an obvious decrease in the latency to the platform, compared with saline treatment (HIBD + TsI: *n* = 11, *p* < 0.05 vs. HIBD + saline; Fig. 2a).

The results from probe test showed that spatial memory retrieval was obviously impaired in HIBD rats since they spent much less time in the quadrant where the hidden platform was previously located (sham: *n* = 10, 28.8 ± 2.7 s; sham + TsI: *n* = 10, 26.4 ± 1.8 s, *p* > 0.05 vs. sham; HIBD + saline: *n* = 10, 14.5 ± 3.6 s, *p* < 0.01 vs. sham; Fig. 2b). As expected, TsI treatment significantly increased the time spent in the target quadrant compared with the HIBD group (HIBD + TsI: *n* = 11, 24.0 ± 2.1 s, p < 0.05 vs. HIBD + saline, *p* > 0.05 vs. sham; Fig. 2b). Additionally, the results of latency to cross the location of hidden platform (sham: *n* = 10, 10.2 ± 3.3 s; sham + TsI: n = 10, 12.8 ± 4.8 s, *p* > 0.05 vs. sham; HIBD + saline: *n* = 10, 43.8 ± 7.0 s, *p* < 0.01 vs. sham; HIBD + TsI: *n* = 11, 15.8 ± 3.4 s, *p* > 0.05 vs. sham, *p* < 0.01 vs. HIBD + saline; Fig. 2c) and the number of crossing the location of hidden platform (sham: *n* = 10, 3.0 ± 0.4; sham + TsI: *n* = 10, 2.1 ± 0.4, *p* > 0.05 vs. sham; HIBD + saline: *n* = 10, 0.8 ± 0.3, *p* < 0.01 vs. sham; HIBD + TsI: *n* = 11, 2.5 ± 0.3, *p* > 0.05 vs. sham, *p* < 0.01 vs. HIBD + saline; Fig. 2d) further confirmed that spatial memory retrieval was impaired after HIBD, and TsI treatment succeeded in preventing this impairment.

**Fig. 3:**
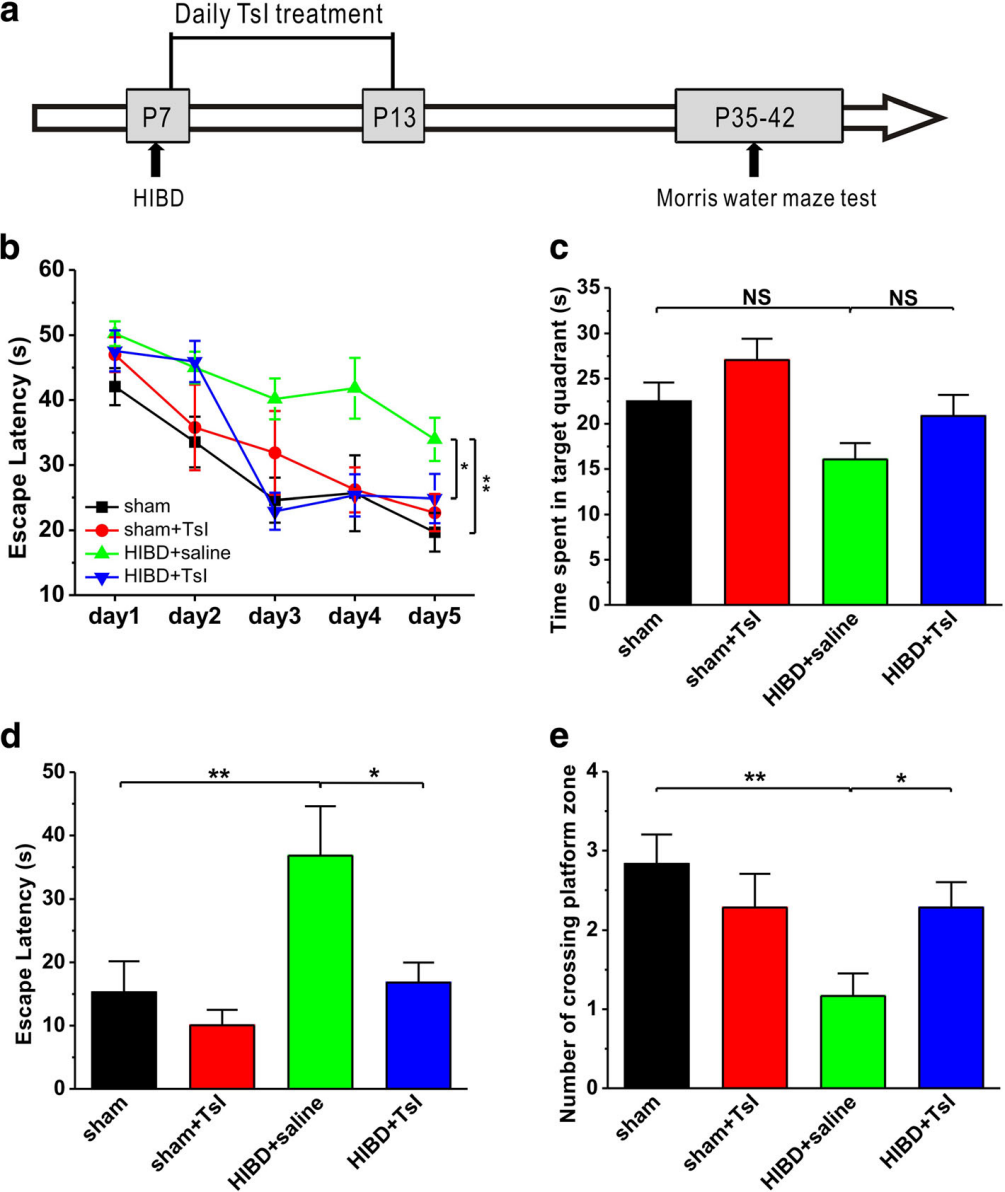
TsI treatment immediately after HIBD alleviates spatial learning and memory deficits in neonatal HIBD rats. **a** The flowchart illustrates the experimental protocols of TsI treatments and water maze test. Rats received TsI treatment (5 mg/kg per day, i.p.) seven times after HIBD model established, and the first treatment of TsI was conducted immediately after HIBD. The Morris water maze test was performed from postnatal day 35 to 42 (P35–42). **b** The average escape latency to the hidden platform location is plotted for each spatial learning day in the Morris water maze task. **c**-**e** Bar graph showed the time spent in the hidden platform-located quadrant **c**, the latency of first time to the hidden platform location (**d**) and the number of entries into the platform zone (**e**) during the probe test with absence of the hidden platform, which is conducted 24 h after the last learning trial. Data was expressed as mean ± SEM. *p < 0.05, **p < 0.01

To further determine the therapeutic effects of TsI on HIBD, we treated the rats with TsI daily for 7 days and the first injection was carried out immediately after HIBD. Four weeks after HIBD, the Morris water maze test was performed. As shown in Fig. 3, the protective effects of TsI on spatial learning and memory in HIBD rats were similar to those that found in pretreatment of TsI, as reflected by an obvious decrease in the latency to the platform, compared with saline treatment (HIBD + TsI: *n* = 7, *p* < 0.05 vs. HIBD + saline; Fig. 3b) during spatial training period. Although the time spent in the target quadrant among these groups was no significant difference (sham: *n* = 6, 22.5 ± 2.1 s; sham + TsI: *n* = 7, 27.1 ± 2.4 s, *p* > 0.05 vs. sham; HIBD + saline: *n* = 6, 16.1 ± 1.8 s, *p* > 0.05 vs. sham; HIBD + TsI: *n* = 7, 20.9 ± 2.3 s, *p* > 0.05 vs. sham, *p* > 0.05 vs. HIBD + saline; Fig. 3c), the latency to cross the location of hidden platform (sham: *n* = 6, 15.3 ± 4.9 s; sham + TsI: *n* = 7, 10.1 ± 2.4 s, *p* > 0.05 vs. sham; HIBD + saline: *n* = 6, 36.8 ± 7.8 s, *p* < 0.01 vs. sham; HIBD + TsI: *n* = 7, 16.8 ± 3.2 s, *p* > 0.05 vs. sham, *p* < 0.05 vs. HIBD + saline; Fig. 3d) and the number of crossing the location of hidden platform (sham: *n* = 6, 2.8 ± 0.4; sham + TsI: n = 7, 2.3 ± 0.4, *p* > 0.05 vs. sham; HIBD + saline: *n* = 6, 1.2 ± 0.3, *p* < 0.01 vs. sham; HIBD + TsI: *n* = 7, 2.3 ± 0.3, *p* > 0.05 vs. sham, *p* < 0.05 vs. HIBD + saline; Fig. 3e) showed that spatial memory retrieval was impaired after HIBD, and TsI treatment succeeded in preventing this impairment.

Taken together, these results suggest that systemic administration of TsI either pretreatment or immediately after HIBD is able to prevent the HIBD-induced impairment of spatial learning and memory.

### TsI reduces HIBD-induced neuron loss

**Fig. 4:**
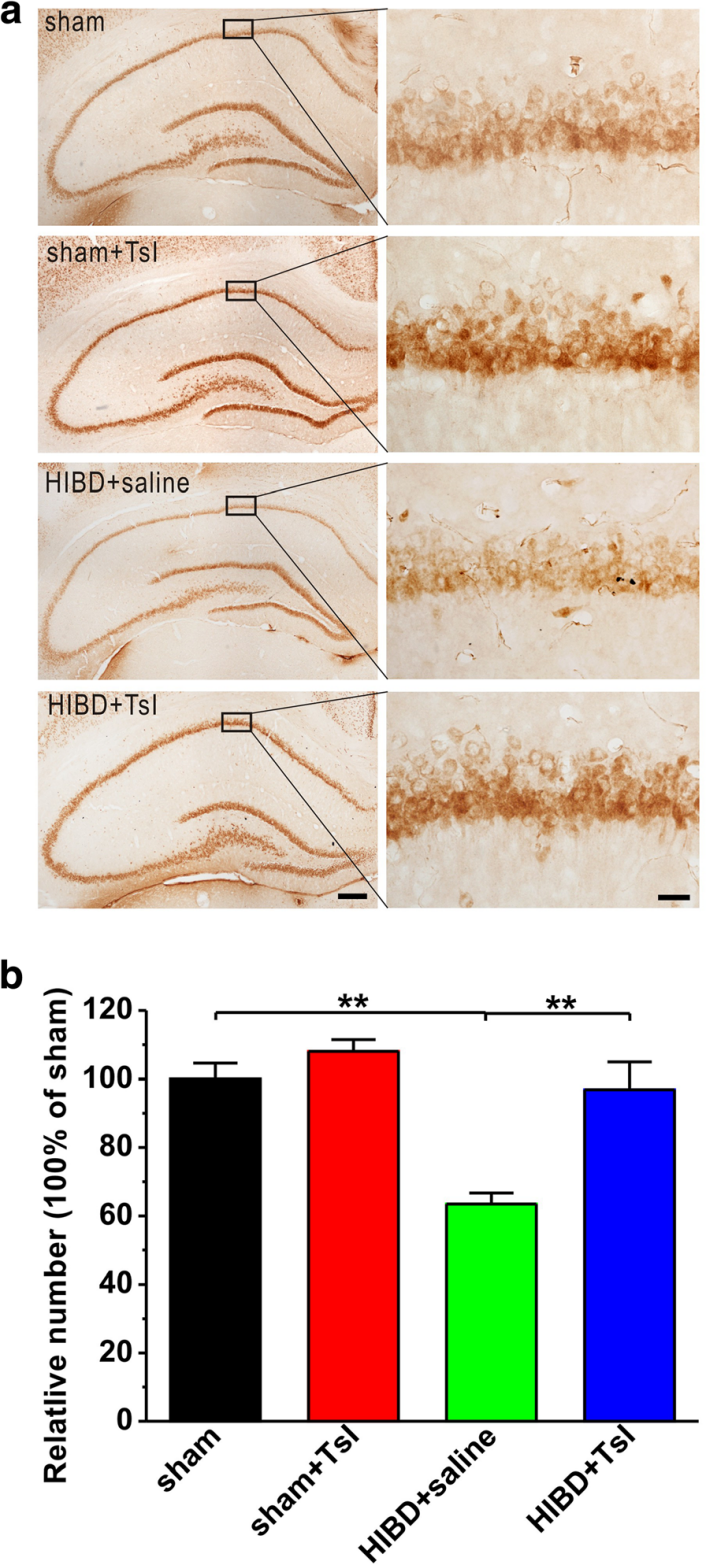
Pretreatment of TsI rescues the decrease of NeuN-immunoreactive neurons in the hippocampal CA1 region of neonatal HIBD rats. **a** Representative photomicrographs of different treatment. Scale bar = 400 μm for left panel and 50 μm for right panel. **b** Bar graph summarizing the relative number of NeuN-immunoreactive neurons in the CA1 region of hippocampus. Data was expressed as mean ± SEM. **p < 0.01

The number of NeuN-immunoreactive neurons was examined to confirm the neuroprotective effects of TsI on pyramidal neurons after hypoxic-ischemic insult in the CA1 area of hippocampus. The results showed that the number of NeuN-immunoreactive neurons in the CA1 area dramatically decreased in the HIBD group compared with the sham group, and administration of TsI significantly suppressed the decrease of NeuN-immunoreactive neurons (sham: *n* = 4; sham + TsI: *n* = 4, 108.0 ± 3.5% sham, *p* > 0.05 vs. sham; HIBD + saline: *n* = 6, 63.5 ± 3.2% sham, *p* < 0.01 vs. sham; HIBD + TsI: *n* = 5, 96.9 ± 8.1% sham, *p* > 0.05 vs. sham, *p* < 0.01 vs. HIBD + saline; Fig. 4).

### TsI reverses the decreased antioxidant and the increased pro-oxidant in HIBD rats

**Fig. 5:**
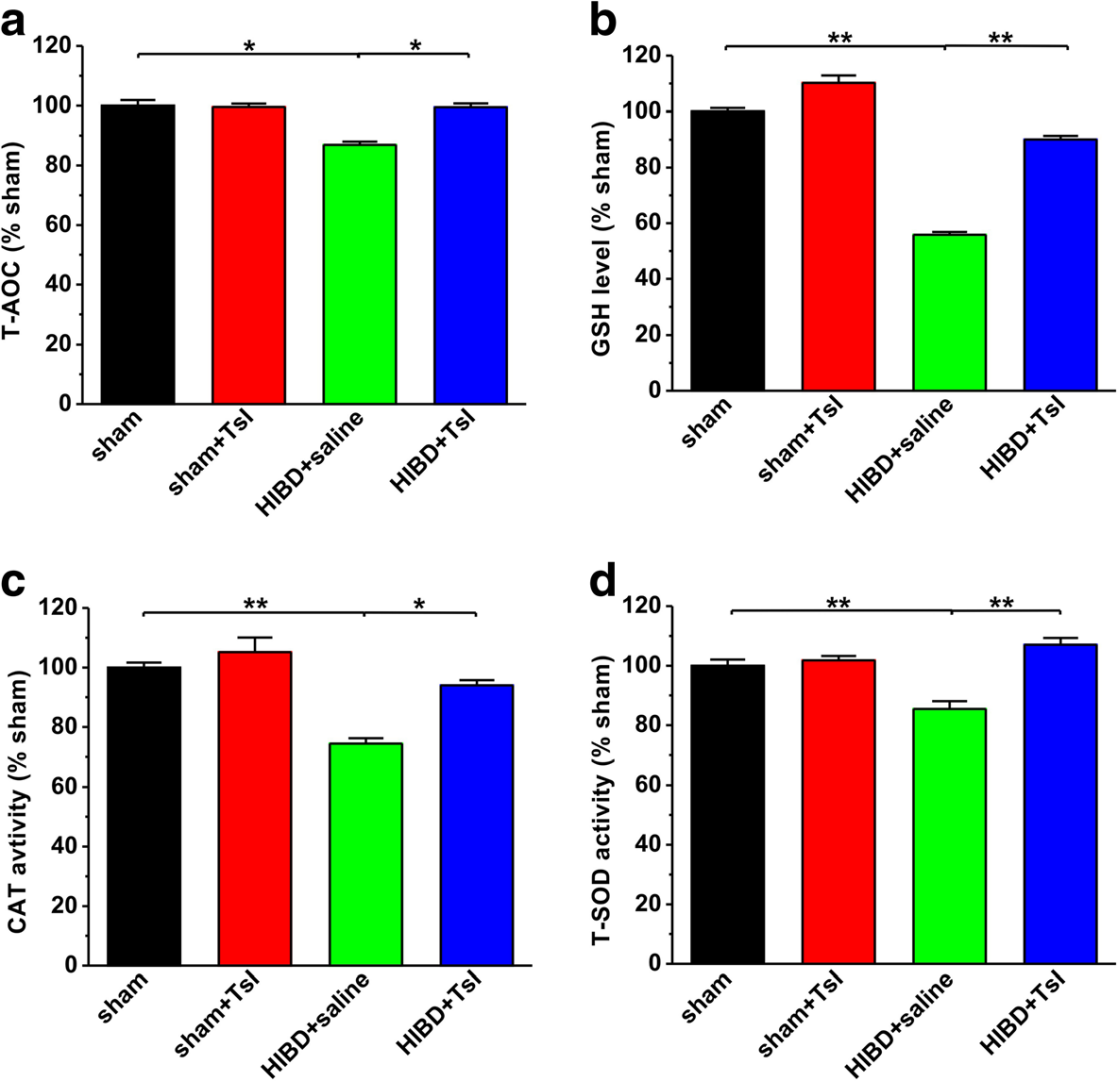
Pretreatment of TsI restores the decreased antioxidants in the brain of HIBD rats. HIBD results in a significant reduction in antioxidants including T-AOC **a**, GSH **b**, CAT **c** and T-SOD **d**, whereas TsI treatment restores these antioxidants to control level. The results were presented as the mean ± SEM. *p < 0.05, **p < 0.01

It has been well known that oxidative injury plays an important role in pathogenesis of HIE [6], and TsI has been shown to exert antioxidant action in various experimental models both in vitro [33, 34] and in vivo [35, 36]. We therefore wanted to determine whether the reduction of neuron loss in HIBD rats treated with TsI could be attributed to its suppressing effect on oxidative stress. As shown in Fig. 5, a significant decrease in the production of antioxidants including T-AOC, GSH, CAT and T-SOD was observed in HIBD group. However, compared with saline treatment, TsI dramatically rescued the T-AOC (sham: *n* = 11, sham + TsI: n = 11, 99.5 ± 2.3% sham, *p* > 0.05 vs. sham; HIBD + saline: *n* = 12, 86.9 ± 2.1% sham, *p* < 0.05 vs. sham; HIBD + TsI: *n* = 13, 99.4 ± 2.5% sham, *p* > 0.05 vs. sham, p < 0.05 vs. HIBD + saline; Fig. 5a), GSH (sham: *n* = 8, sham + TsI: n = 8, 110.2 ± 13.3% sham, *p* > 0.05 vs. sham; HIBD + saline: n = 8, 55.8 ± 5.2% sham, *p* < 0.01 vs. sham; HIBD + TsI: n = 8, 90.1 ± 6.3% sham, *p* > 0.05 vs. sham, p < 0.01 vs. HIBD + saline; Fig. 5b), CAT (sham: *n* = 9, sham + TsI: *n* = 10, 105.1 ± 9.8% sham, *p* > 0.05 vs. sham; HIBD + saline: n = 10, 74.4 ± 3.7% sham, *p* < 0.01 vs. sham; HIBD + TsI: *n* = 12, 94.0 ± 3.5% sham, *p* > 0.05 vs. sham, *p* < 0.05 vs. HIBD + saline; Fig. 5c) and T-SOD (sham: *n* = 8, sham + TsI: *n* = 7, 101.8 ± 3.1% sham, *p* > 0.05 vs. sham; HIBD + saline: *n* = 8, 85.4 ± 5.2% sham, *p* < 0.01 vs. sham; HIBD + TsI: *n* = 7, 107.0 ± 4.6% sham, *p* > 0.05 vs. sham, *p* < 0.01 vs. HIBD + saline; Fig. 5d) activity following HIBD in the brain.

**Fig. 6:**
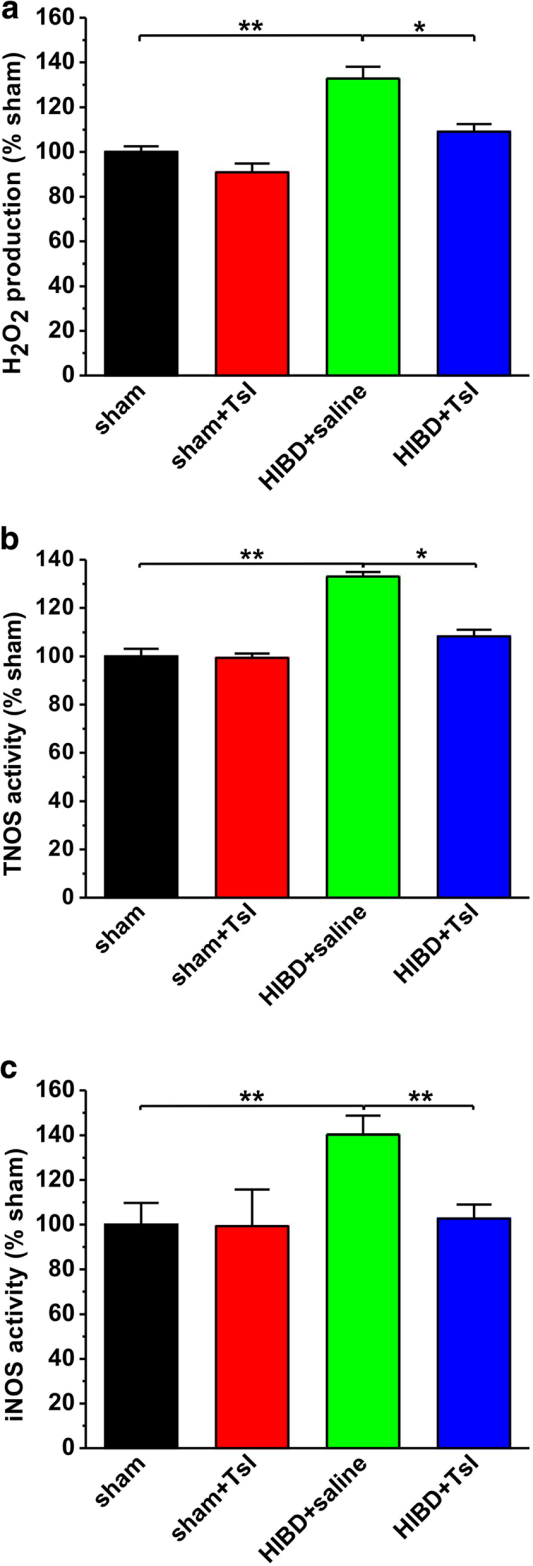
Pretreatment of TsI restores the increased pro-oxidants in the brain of HIBD rats. HIBD results in a significant increase in pro-oxidants including H_2_O_2_ **a**, TNOS **b** and iNOS **c**, whereas TsI treatment restores these pro-oxidants to control level. The results were presented as the mean ± SEM. *p < 0.05, **p < 0.01

Meanwhile, as shown in Fig. 6, a significant increase in the production of pro-oxidants including H_2_O_2_, TNOS and iNOS was observed in HIBD group. As expected, TsI dramatically suppressed the increase of H_2_O_2_ (sham: *n* = 10, sham + TsI: *n* = 10, 90.9 ± 7.9% sham, *p* > 0.05 vs. sham; HIBD + saline: *n* = 12, 132.7 ± 10.5% sham, *p* < 0.01 vs. sham; HIBD + TsI: n = 12, 109.0 ± 6.8% sham, p > 0.05 vs. sham, p < 0.05 vs. HIBD + saline; Fig. 6a), TNOS (sham: *n* = 11, sham + TsI: n = 9, 99.4 ± 3.6% sham, p > 0.05 vs. sham; HIBD + saline: n = 11, 133.0 ± 3.7% sham, p < 0.01 vs. sham; HIBD + TsI: *n* = 13, 108.3 ± 5.6% sham, p > 0.05 vs. sham, p < 0.05 vs. HIBD + saline; Fig. 6b) and iNOS (sham: n = 10, sham + TsI: n = 10, 99.4 ± 16.3% sham, p > 0.05 vs. sham; HIBD + saline: n = 12, 140.3 ± 8.4% sham, p < 0.01 vs. sham; HIBD + TsI: *n* = 14, 102.7 ± 6.3% sham, p > 0.05 vs. sham, *P* < 0.01 vs. HIBD + saline; Fig. 6c) following HIBD in the brain, compared with saline treatment. These results indicate a powerful antioxidative capacity of TsI in HIBD rats.

## Discussion

In the present study, we demonstrate that TsI, a compound purified from the Chinese herb Danshen, suppresses the decrease of pyramidal neurons in the CA1 area of hippocampus, and alleviates the impairment of motor and spatial learning and memory functions in the neonatal rats subjected to HIBD. We further confirm that the beneficial effects of TsI on HIBD rats are associated with the increase of antioxidant activity. We have therefore provided evidence that TsI may suppress HIBD-induced oxidative stress and subsequently block the loss of pyramidal neurons in the CA1 area of hippocampus, thereby ameliorating motor and cognitive decline.

The dried root of Salvia miltiorrhiza, called Danshen in China, has been used effectively for the treatment of many diseases, especially ischemic cardiovascular disease in aging people for over 1000 years [12]. Previous study has indicated that Danshen is able to dilate cerebral arteries, increase cerebral blood flow, inhibit oxidative stress and reduce neuronal death induced by ischemia [14]. In addition, a growing body of evidence has shown that the major lipophilic diterpenes derived from Danshen extract, such as CTs, DTsI, TsI, TsIIA and TsIIB, have neuroprotective effects against transient ischemic damage in different adult animal models including gerbils [35], rats [24] and mice [22, 23]. Recent studies have reported that TsIIA treatment is significantly protective for hypoxia-ischemia in the immature rat [25, 26]. Similar to TsIIA, we here found that treatment with TsI markedly suppressed the loss of pyramidal neurons induced by hypoxia-ischemia in the CA1 region of hippocampus (Fig. 4). However, the dose of TsI we used in the present study was 5 mg/kg/day, which is much lower than TsIIA (10 mg/kg/day) used in previous study [26], suggesting that TsI may be more effective than TsII for treating HIBD.

The neuronal loss induced by hypoxia-ischemia is usually associated with the deficits of motor and cognitive functions both in animal models and in patients [37–39]. Excitedly, recent study has revealed that TsI is able to ameliorate the learning and memory impairments induced by a GABA_A_ receptor agonist diazepam, or an NMDA receptor antagonist MK-801, in adult mice [40]. However, so far no study to examine the influence of TsI on motor and learning and memory functions in immature animals after hypoxia-ischemia. In the current study, we found that TsI could rescue myodynamia and motor deficits (Fig. 1), and alleviate the spatial learning and memory impairments (Figs. 2 and 3) in neonatal HIBD model rats. Combined with previous report [40], we can therefore conclude that TsI has the ability to ameliorate the learning and memory impairments in both adult and neonatal animals.

Although HIBD is a complex multifactorial disease, a growing body of evidence has shown that oxidative damage to vital cellular structures plays a critical role in the pathogenesis of brain damage in both the immature and mature nervous system [6, 41, 42]. Several studies have indicated that TsI is able to exert antioxidative and antiapoptotic effects in cellular and mouse model of Parkinson’s disease (PD) [18, 19, 43]. In full agreement with these findings, we here found that treatment with TsI markedly reversed the decrease of antioxidants and the increase of pro-oxidants induced by HIBD in neonatal rats (Figs. 5 and 6). The exact underlying cellular and molecular mechanisms remain to be determined, but may be at least in part due to the up-regulating effects of TsI on nuclear factor erythroid-2-related factor 2 (Nrf2), since previous studies have supported that the antioxidation of TsI is involved in Nrf2 signaling pathway both in vitro and in mouse model of PD [43, 44]. Thus, future experiments examining Nrf2 signaling pathway in neonatal HIBD animals with or without TsI treatment will help determine whether the therapeutic effects of TsI in neonatal HIBD rats can be attributed to its up-regulation of Nrf2. Furthermore, some studies have reported that the activation of nuclear factor kappa B (NF-κB) promotes cell survival via attenuating reactive oxygen species (ROS), such as NOS, H_2_O_2_, et al. [45, 46], and increasing the expression of antioxidant proteins, such as GSH, SOD, catalase, and so on [47–49]. Here, we found that TsI significantly promoted the expression of antioxidant proteins and suppressed the expression of pro-oxidants (Figs. 5 and 6). However, further studies would be necessary to elucidate the full mechanism whether TsI produces neuroprotection via activating NF-κB signaling pathway.

## Conclusions

In summary, these data provide the first evidence that TsI treatment suppresses HIBD-induced neuronal death and oxidative stress, thereby ameliorating myodynamia and motor abilities as well as spatial learning and memory in neonatal rats after HIBD, an animal model of HIE in patient, suggesting that TsI may represent a potentially effective therapeutic drug for HIE.

## Abbreviations

CAT: Catalase
CTs: Cryptotanshinone
DMSO: Dimethyl sulfoxide
DTsI: Dihydrotanshinone I
GST: Glutathione
H_2_O_2_: Hydrogen peroxide
HIBD: Hypoxic-ischemic brain damage
HIE: Hypoxic-ischemic encephalopathy
iNOS: Inducible nitric oxide synthase
NF-κB: Nuclear factor kappa B
Nrf2: Nuclear factor erythroid-2-related factor 2
ROS: Reactive oxygen species
T-AOC: Total antioxidant capacity
T-NOS: Total nitric oxide synthase
TsI: Tanshinone I
TsII: Tanshinone II
T-SOD: Total superoxide dismutase
